# Studying the impact of a medication use evaluation for polymedicated older patients by the community pharmacist (SIMENON): study protocol

**DOI:** 10.1186/s12913-018-3440-z

**Published:** 2018-08-08

**Authors:** J. Wuyts, J. Maesschalck, I. De Wulf, K. Foubert, K. Boussery, J. De Lepeleire, V. Foulon

**Affiliations:** 1 Department of Pharmaceutical and Pharmacological Sciences, KU Leuven, Herestraat 49 O&N2, Box 521, 3000 Leuven, Belgium; 2 Association of Belgian Pharmacies (APB), Archimedesstraat 11, 1000 Brussels, Belgium; 3 Pharmaceutical Care Unit, Faculty of Pharmaceutical Sciences, Ghent University, Ghent, Belgium; 4 Department Public Health and Primary Care, ACHG, KU Leuven, Leuven, Belgium

**Keywords:** Aged, Medication review, Drug-related problems, Medication use, Community pharmacist

## Abstract

**Background:**

Aged polymedicated patients are particularly vulnerable for drug-related problems. A medication review aims to optimize the medication use of patients and improve health outcomes. In this study, the effect of a pharmacist-led medication use review is investigated for polymedicated ambulatory older patients with the aim of implementing this pharmaceutical care intervention across Belgium.

**Methods:**

This article describes the study protocol of the SIMENON study and reports the results of the feasibility study, which aimed to test and optimize this study protocol. In the SIMENON intervention study, 75 Belgian community pharmacies each recruit 12 patients for a medication use review. For each patient, the identified drug-related problems and subsequent interventions are registered using the PharmDISC classification. In a subset of Dutch speaking patients, a pretest-posttest single group design is used to measure the impact of this review on patient related outcomes using questionnaires. The main outcome of the study is the type and number of drug-related problems and related interventions. A second outcome is the impact of the medication use review on adherence, objectively measured with dispensing data. Evolution in medication related quality of life is another outcome, measured with the Living with Medicines Questionnaire version 3. Other patient reported outcomes include adherence, self-management, patient satisfaction, fall incidents and use of emergency healthcare services.

**Discussion:**

The findings of this study can provide data on the effectiveness of a medication use review in the Belgian primary care setting. Furthermore, it will provide insights in which patients benefit most of this intervention and therefore facilitate the implementation of medication review in Belgium.

**Trial registration:**

ClinicalTrials.gov NCT03179722. Retrospectively registered 7 June 2017.

## Background

Aging populations, increase in chronic conditions and polypharmacy are challenging healthcare systems worldwide. In Belgium 17.9% of the population is aged 65 or more and at least 19% of these patients are polymedicated [1]. Polymedication entails a risk for drug-related problems such as non-adherence, drug interactions, adverse drug events and hospitalizations [2]. Approximately 6 to 17% of acute hospitalizations in older patients are drug-related [3, 4]. In Belgium 42.000 medication-related hospital admissions are potentially preventable per year and could save 200 million euros on a yearly basis [3–5].

A medication review is defined as “a structured evaluation of a patient‘s medicines with the aim of optimizing medicines use and improving health outcomes. This entails detecting drug-related problems and recommending interventions” [6]. Depending on the sources of information used in the review, three types are distinguished: a basic, intermediate and advanced medication review [6]. Evidence exists that medication reviews can improve outcomes such as adherence, appropriateness of prescribing and emergency department contacts [7–9]. However the evidence for an impact on the number of hospitalizations is contradicting [7, 10].

The rising trend of medication review services in healthcare is undeniable [11]. In countries such as Australia, The Netherlands, The United Kingdom, Portugal, Denmark and Switzerland medication reviews are a nationally implemented reimbursed healthcare service [11]. Contrasting, the target population for an intermediate medication review differs across countries and further research should identify and target patients who would benefit most of a review [8, 11].

The implementation of clinical services by pharmacists in Belgium is also progressing. Since 2013, Belgian pharmacists offer an advanced pharmaceutical care service for new asthma patients. Moreover the federal government has funded research on advanced medication reviews in Belgian nursing homes [12]. Similarly, the Association of Belgian Pharmacies has now taken initiatives to investigate and implement intermediate medication reviews in primary care. For that purpose, a consortium with three Belgian universities was set up to prepare and research the implementation of this type of review, further referred to as medication use review (MUR), in Belgian community pharmacies.

In a first step, a feasibility study was conducted to test the medication review six step process. The protocol, study materials and measures were optimised based on the feedback of these participants. In a second step, the objective is to Study the Impact of a Medication use EvaluatioN by the cOmmuNity pharmacist (SIMENON study) in older polymedicated patients. Other members of the consortium will focus on the added value of the standard application of the GheOP^3^S tool^1^ (to evaluate appropriateness of prescribing) and multidisciplinary case conferences during the medication review process [13]. Furthermore, thorough research on the implementation of the service will be performed using the RE-AIM model [14].

## Methods/design

This study protocol is developed in line with SPIRIT recommendations for reporting clinical trial protocols [15].

### Design, setting and study period

The SIMENON study is an intervention study with a pretest-posttest single group design. The intervention is performed by community pharmacists in the primary care setting. Patients are followed over a period of 9 months. Patient recruitment was planned from November 2016 until March 2017.

### Pharmacy

Pharmacies in the three regions of Belgium (Flanders, Brussels and Wallonia) could participate. Recruitment of Belgian pharmacies occurred in the fall of 2016 via national and local pharmaceutical magazines. No inclusion criteria were set but participation to the study workshop (see 6. Education program) and a signed collaboration agreement are required. Multiple pharmacists within one pharmacy can perform medication reviews. Participation comprises of performing twelve medicines use reviews, registering the findings in a webtool and delivering medication refill records of the participating patients to the research team.

### Patient

Four patient inclusion criteria are set: patients should be 70 years or older, use five or more drugs chronically (both prescription or non-prescription drugs) and live in the ambulatory setting. The age limit is set at 70 years. Given the high population of patients aged 65 or older in Belgium (approximately 2 million people), the researchers wanted to further limit the size of the eligible study population. In line with the aging population, several projects have been conducted in primary care in Belgium which focused on patients aged 70 or more instead of 65 [16]. Finally, the patient should obtain his medication from this pharmacy on a regular basis. Patients can be Dutch or French speaking. Dutch patients are also invited by their pharmacist to participate to questionnaires for a pretest-posttest measurement. Having a medication overview or the presence of a carer or home nurse are no exclusion criteria since the patient remains involved in his medication use and/or medication experiences. Carers are allowed to accompany the patient for the medication review.

### Recruitment and consent

Based on the suggestions in the feasibility study (see 12. Feasibility study), pharmacists are required to approach each patient who meets the inclusion criteria on fixed inclusion days to avoid selection bias. Upon informed consent, Dutch speaking patients are proposed to fill out questionnaires (see 9. Data collection). Patients can indicate if they prefer to be contacted by the research team either by phone, by e-mail or by letter to complete the surveys.

### Intervention

The intervention consists of a medication use review. In this intermediate medication review both a patient interview and medication refill records are used as information sources to identify drug-related problems with a specific focus on the medicines use of the patient [6]. Table 1 shows the six components of the intervention which is based on the Systematic Tool to Reduce Inappropriate Prescribing (STRIP) tool [17]. A checklist is available for participants to ensure the quality of the medication reviews (available upon request). The feasibility study confirmed the applicability of this six step process and estimated a workload of 120 min per review.

**Table 1:** The six-step process of a medication review [17]. The time points of data collection for the SIMENON study are indicated. Both the community pharmacists (CP) and the researchers (R) collect data at different time points

	Component	Time point	Data collection	
Medication review	1 Patient recruitment	T_-1week_	R	
	2 Preparation			CP
	3 Patient interview	T_0_		CP
	4 Pharmacotherapeutic analysis			
	5 Discussing medication list	T_2weeks_		CP
		T_3weeks_	R	
	6 Follow up	T_6weeks_		CP
		T_12weeks_	R	

Upon patient recruitment, the patient is invited for a pharmacotherapeutic anamnesis also referred to as a patient interview [17]. This interview can be either on appointment, during the next visit or immediately upon consent. Patients are encouraged to bring their medication (brown bag method) to this interview and list their current medication. The anamnesis should preferably take place in the pharmacy, if possible in a private area. In exceptional circumstances such as patient immobility, the pharmacist can perform the anamnesis at the patients’ home. In preparation for the anamnesis, the pharmacist consults the medication refill records to review adherence and check drug interactions.

The aim of the patient interview itself is to gain insight in the pharmacotherapy of the patient. Data on medication use, medication knowledge but also experiences are gathered. Additional questions relate to non-medication treatments, lifestyle, living situation, allergies, functional status, hospitalization and fall incidents.

Subsequently, the pharmacist performs a pharmacotherapeutic analysis to identify drug-related problems with particular focus on the medication use. Next, priorities are set and interventions are prepared. The minimal output of a medication review is a medication overview for the patient. This overview comprises the current chronic medication of the patient, both prescribed and non-prescribed. Aside from the name of the medication, also the frequency and time of intake and remarks are registered in this overview.

In the fifth step, the pharmacist and patient reconvene to discuss and execute the interventions. The pharmacist runs through the medication overview with the patient and checks if everything is understandable for the patient. It is recommended to perform this second consultation within 2 weeks after the anamnesis. Finally, in the follow up conversation the pharmacist investigates if the undertaken actions were useful and investigates if new problems have emerged or new interventions are required. If necessary, the medication overview is updated. It is recommended to perform this follow up a month after the second patient-pharmacist conversation.

Although the medication use review is initiated by the community pharmacist, collaboration with the general practitioner (GP) is encouraged. For instance by informing the GP before the anamnesis, by actively discussing the pharmacotherapy of the patient during the analysis or by sharing the medication overview of the patient. The feasibility study uncovered there was no consensus on how the general practitioner should be involved in the review process. There was no support to organize interdisciplinary case conferences for each patient, nor to inform the GP before each review. Depending on the drug-related problems that are discovered, discussion with the GP or patient referral may be necessary.

### Education program

The educational program for participating pharmacists consists of five components: an information session, workshop, toolbox, documentation and an intervision session (Table 2). All sessions are organized in fivefold across Belgium. The feasibility study provided insights in the training needs of pharmacists in Belgium and was used to optimize this educational program.

**Table 2:** Overview of the topics in the educational program of the SIMENON study

Education program	Topic			
	Study procedure	Background and evidence based info	Communication skills and patient inclusion	Multidisciplinary collaboration
Information session	X			
Workshop	X		X	X
Toolbox		X		
Documentation	X		X	X
Intervision session	X		X	X

In the first information session interested pharmacists are informed about the aims of the SIMENON study, the procedure of a medicines use review and are given the opportunity to ask questions. In the subsequent obligatory interactive workshop of 2 hours, participants are familiarized with a MUR. The research team of KU Leuven demonstrates a MUR using a simulation patient. Attention is drawn to the drug-related problems that should be detected and its classification (see 7. Outcomes) and to communication skills for patient and GP conversations.

The toolbox supports participants in the analysis phase by providing background information and evidence-based references and websites. The following six topics are addressed, based on the feedback in the feasibility study: geriatric patients and characteristics, drug interactions and side effects, adherence, drug use including time of intake and a written example of a medication review.

The following documentation is available for the community pharmacist: an overview of the steps of a medication review including a checklist, a letter for the GP with study information and a referral letter. For the patients, an information leaflet with informed consent form is foreseen as well as a blank medication list and a brown bag. Finally, an intervision moment is organized halfway in the project for participants to exchange experiences, feedback and tips and tricks for patient inclusions.

### Outcomes

The main outcome of the study is the type and number of drug-related problems and related interventions registered by pharmacists using the validated PharmDISC^2^ classification tool (a process measure) [18]. A second outcome is the impact of the medication review on adherence, objectively measured with medication refill data. A third outcome is the medication related quality of life, measured with the Living with Medicines Questionnaire version 3 (LMQ) [19]. Other patient related outcomes are adherence, self-management, patient satisfaction, use of emergency healthcare services and fall incidents. These outcomes are measured via the pre-post study design using patient questionnaires.

### Data collection

An overview of the data collection strategies is shown in Table 3. Four types of quantitative data are collected: drug-related problems and interventions, medication refill records, patient questionnaires and descriptive pharmacist information. If patients would withdraw consent, no additional data is registered but existing data is preserved.

**Table 3:** Overview of the data collected for the SIMENON study

Data collection	A. Drug-related problems & interventions	B. Adherence	C. Questionnaires	D. Descriptive pharmacist information
Subject	Patient	Patient	Patient	Pharmacist
Collector	Pharmacist	Pharmacist	Researcher	Pharmacist
Sample size	900 patients	900 patients	140 patients	75 pharmacies
Method	Questionnaire: webtool	Medication refill records	Questionnaire: by letter, by phone or by e-mail	Questionnaire
Frequency	Four times	Once	Three times	Once
Timeframe	During the medication review process	• 9 months before • 9 months after the review	• 1 week before the review • 3 weeks after the review • 12 weeks after the review	Before start of the study
Content	• Administrative and medical data • DRPs and interventions • Duration • GP contacts	Medication refill records	• Adherence • Self-management • Polypharmacy burden • Satisfaction • Demographic data • Use of emergency healthcare services • Fall incidents	• Pharmacy and Pharmacist characteristics • Pharmacist experiences

For each patient receiving a review, an online registration form is completed by the pharmacist via a webtool (available upon request). Pharmacists are required to login to use this webtool and only coded patient information can be registered. Drug-related problems and interventions are registered throughout the medication review process using the PharmDISC classification system [18]. Permission was given by the authors to translate and use the instrument. Participating pharmacists are given a manual with explanation and examples to ensure the quality of the classification. Medication refill data is collected of all participants in the study to estimate adherence before and after the medication review. Data is collected from 9 months before until 9 months after the review. Finally, all participating pharmacists are required to fill in a questionnaire related to pharmacy and pharmacist characteristics.

The effectiveness of a medication use review is evaluated using patient reported outcomes. Patients are surveyed at three time points by the research team of KU Leuven (Table 4). Based on the preferences of the patient the questionnaires can be completed by e-mail, by phone or by mail. These surveys allow an insight in patient experiences. Table 4 provides an overview of instruments and the time points of data collection. Permission was given by all authors to use the instruments. The ‘Living with Medicines Questionnaire’ and the ‘Patient Satisfaction with Pharmacist Services Questionnaire’ were translated by the researchers in accordance with ISPOR guidelines [20].

**Table 4:** Patient related outcome measures. Data is collected 1 week before the patient interview (T_-1w_), 3 weeks later (T_3_) and 12 weeks after the patient interview (T_12w_)

Subject	Instrument	Authors	T_-1w_	T_3w_	T_12w_
Adherence	Probabilistic Medication Adherence Scale (ProMAS)	Kleppe et al. 2015 [21]	X		X
Self-management	Patient Activation Measure (PAM)	Hibbard et al. 2004 [22]	X		X
Polypharmacy burden	Living with Medicines Questionnaire (LMQ)	Krska et al. 2014 [23]	X	X	X
Satisfaction	Patient Satisfaction with Pharmacist Services Questionnaire (PSPSQ) 2.0	Sakharkar et al. 2015 [24]		X	
Demographic data	Living with Medicines Questionnaire (LMQ)	Krska et al. 2014 [23]	X	X	X
Use of emergency healthcare services	/		X		X
Fall incidents	/		X		X

### Data management

Patient confidentially is ensured through patient coding during data registration by the treating community pharmacist. Data obtained from the questionnaires are also coded by the researchers. The study database only contains coded data. Data cleaning and analysis is performed by the research group of KU Leuven. All parties of the consortium have access to the final database as stated in the collaboration agreement. Sharing of anonymized individual clinical trial participant-level data is possible for research purposes which are in line with the initial aim of data collection. Data is disseminated through publication in peer reviewed international journals.

### Data analysis

First, descriptive statistics will be used to characterize the study population using demographic parameters, to give an overview of drug-related problems, interventions and level of implementation. Secondly, adherence will be calculated using two methods for the analysis of refill data: the medication possession ratio as well as proportion of days covered. The impact will be measured by comparing data before and after the intervention using appropriate statistical tests for paired data. Subsequently the correlation between objective measures for adherence (medication refill records) and subjective measures (PROMAS score) will be investigated [21]). In addition, the results of these adherence measures will be compared with the registered drug-related problems and interventions. This provides insight in the number of (un)detected adherence problems and the effectiveness of a medication review and interventions to improve adherence.

Third, patient questionnaires will be scored in accordance with the described methods of the original authors. The impact of the intervention will be explored using appropriate statistical tests for paired data. Subgroup analysis will compare the baseline scores in the three types of data collection (letter, phone or email) to ensure the quality of the data.

A final analysis aims to identify patients with a high risk of drug-related problems by investigating the impact of variables on the number of drug-related problems detected by pharmacists. These variables have been defined a priori based on scientific research as well as patient criteria for a medication review in countries abroad. Both patient related factors, process factors and pharmacy related factors will be explored. All analysis will be performed using the statistical program SPSS. A per protocol analysis and a listwise deletion will be applied.

### Sample size

The study aims to approach 75 pharmacies in Belgium, approximately half in Flanders and half in Wallonia. Each pharmacy in turn includes 12 patients. This brings the total number of participants to 900 (Table 5). The primary aim of this study is to describe the type and number of drug-related problems and related interventions for each patient. In this way, the primary outcome is a process measure. The secondary outcome is medication adherence. A systematic review by Hatah et al. shows that both an intermediate and advanced medication review by community pharmacists significantly improves adherence [7]. Provided that the pharmacist can improve adherence using a medication review in half of the patients with a low adherence (10% of all older patients) and a significance level of 5% and power of 80%, a sample size of 746 patients is calculated [21, 22]. Taking into account a drop-out rate and missing data (17%, *n* = 154) a sample size of 900 participants is proposed.

**Table 5:** Sample size

Sample size	Medication review	Questionnaires
Number of Belgian community pharmacies	75	35
Number of participating patients per pharmacy	12	4
Total number of participating patients	900	140

For the patient reported outcomes using questionnaires, all Dutch-speaking patients in the Flemish community pharmacies (approximately 35) are approached. It is hypothesized that 2/3 of patients who consent to a medication review also agree to complete the questionnaires. Considering a drop-out rate of 50%, data should be available for 140 patients (Table 5). In this subset, the score on the Living with Medicines Questionnaire is the main outcome. However, since this instrument has not yet been used in an older polymedicated population, no sample size calculation can be performed.

### Feasibility study

In preparation for the SIMENON study, a feasibility study was conducted from March 2016 until September 2016. The aim was to gather insights in the feasibility of the proposed medication review six step process, the protocol of the study (with specific attention to patient inclusion criteria and recruitment) and the content of the educational program. Using purposive sampling, six pharmacists participated, both Dutch and French speaking from different regions. Ethical approval was granted for this study in May 2016 by the Ethics committee of UZ/KU Leuven (S58952 V3) and after that patient inclusion commenced. In total 19 patients were recruited.

Using an iterative process with group discussions and individual contacts, feedback of the participating pharmacists was incorporated in the design of the SIMENON study. Specific feedback was given in relation to the educational program and more specifically the need for background information. No significant adjustments were required for the medication review process itself. Patient recruitment proved to be the most important hurdle. The shared decision was made to work with fixed inclusion days in the pharmacy to minimize selection bias. Finally, the different participants all had different approaches to communicate with the treating physician ranging from no contact, contact before the medication review and contact if necessary in the fourth step of the review process. None of the participants supported the proposal of a multidisciplinary case conference for each patient since the primary focus of the review was medication use rather than appropriateness of prescribing.

### Study registration

This study protocol has been reviewed and approved by the ethics committee UZ/KU Leuven (number S59676 V3) in November 2016. The SIMENON study has been retrospectively registered in June 2017 in the trial database available at www.clinicaltrials.gov (NCT03179722).

### Trial status

Study recruitment commenced on 5 December 2016. In March 2017, the aimed number of included patients was not attained. Therefore, the decision was made to prolong patient inclusions until the end of May and postpone study finalization until March 2018. The final dataset should be collected by October 2018.

## Discussion

In this study intermediate medication reviews are conducted and evaluated. The main outcome of the study is the type and number of drug-related problems and related interventions. A second outcome is the impact of the medication use review on adherence, objectively measured with dispensing data. A third outcome is the impact on the medication related quality of life, measured with the Living with Medicines Questionnaire. This study is in line with similar pharmacist-led interventions worldwide. In Europe alone, initiatives on intermediate medication review have been performed in at least 11 countries [11]. The findings and experiences from these countries have been incorporated in this study protocol.

For one, the target population consists of aged, polymedicated patients. These criteria are in line with the recommended target population for clinical pharmacy services in the Netherlands and UK [23, 24]. This research will provide further insights in the patient population who benefits most of a medication review. For this purpose, also the assistance patients receive in their medicines use, both by professionals, carers and devices such as pill boxes are registered. Secondly attention is drawn to a random patient inclusion in the pharmacy to avoid selection bias. Furthermore, there is an obligatory follow up consultation. In contrast to similar studies, this study not only investigates DRPs and interventions but also focusses on the actual implementation of the interventions [25]. Unlike the study of Holland et al. 2005, this intervention is conducted by trained local community pharmacists with whom the patient has a therapeutic relationship [26]. This real-life context could facilitate the implementation in a later phase. In addition, the feasibility study provided insight in training needs of participants and the feasibility of the study protocol. Another strength of this study is that the outcomes allow insight in both the clinical impact by detecting and resolving drug-related problems as well as the patient perspective on the medication use, medicines experiences and the perceptions on the new service. The systematic review by Loh et al. 2016 has demonstrated there is no evidence that a MR significantly improves overall health related quality of life, possibly due to the generic nature of the instruments used. Therefore, this study is the first to use a medication specific tool, the Living with Medicines Questionnaire, to map the medication related quality of life [19, 27]. In addition, the adherence outcome is both objectively measured as well as using subjective patient questionnaires. A final strength of the study is that it is performed by a consortium of three Belgium universities and the national pharmacy association. This allows a combination of research perspectives.

This study also has several limitations. For one this study is no randomized controlled trial. Although there is no control group, the study provides a detailed description of the drug-related problems and pharmacy interventions. Since many other countries have implemented medication review in the past, this study is on that aspect not very innovative, yet essential since the healthcare system differs across countries and subsequently not all findings are transferable. By using voluntary participation of pharmacists, this creates participating bias. Although the patients should be randomly chosen, it is expected that the most motivated patients who are open to this intervention are included in the study. This also limits the generalizability of the findings. Moreover, the patient follow up is limited to 3 months. Literature suggests this may be rather short to detect an impact on the quality of life [25].

Another limitation of this medication review is that collaborative practice with the general practitioner is not required. Additional input from the GP would indisputably be of added value. However, only a limited amount of initiatives (3/11) in Europe organize case conferences during an intermediate medication review [11]. Moreover, the findings of the feasibility study showed reluctance. Notwithstanding GP collaboration is registered in the webtool and allows subgroup analysis of patients for which multidisciplinary contacts were made.

In conclusion, this study contributes to the evolving role of community pharmacists in Belgium. The findings of this study could provide valuable insights on the drug-related problems that older polymedicated patients experience in Belgium, the patients who would benefit most of a medication review and the value of the community pharmacist in resolving these problems. Therefore, the SIMENON study could significantly support the implementation of medication reviews in Belgium.

## Abbreviations

CP: Community pharmacist
GheOP^3^S tool: Ghent older people’s prescriptions community pharmacy screening tool
GP: General Practitioner
LMQ: Living with medicines questionnaire
MUR: Medication use review
PAM: Patient activation measure
PharmDISC: Pharmacist’s Documentation of Interventions in Seamless Care
PRoMAS: Probabilistic medication adherence scale
PSPSQ: Patient satisfaction with pharmacist services questionnaire
SIMENON: Study the impact of a medication use EvaluatioN by the cOmmunity pharmacist
STRIP: Systematic tool to reduce inappropriate prescribing

## Footnotes

1 GheOP^3^S tool: Ghent Older People’s Prescriptions community Pharmacy Screening tool

2 PharmDISC: Pharmacist’s Documentation of Interventions in Seamless Care
